# Health Literacy of Children and Adolescents with Inflammatory Bowel Disease (IBD) and Parents of IBD Patients—Coping and Information Needs

**DOI:** 10.3390/children11040481

**Published:** 2024-04-17

**Authors:** Kalina Kaul, Stefan Schumann, Cornelia Sander, Jan Däbritz, Jan de Laffolie

**Affiliations:** 1 Department of General Pediatrics and Neonatology, University Children’s Hospital, University Giessen, 35392 Giessen, Germany; kalina.kaul@paediat.med.uni-giessen.de (K.K.); jan.delaffolie@paediat.med.uni-giessen.de (J.d.L.); 2 German Crohn’s Disease and Ulcerative Colitis Association, DCCV, National Association for Chronic Inflammatory Diseases of the Digestive Tract, 10179 Berlin, Germany; csander@dccv.de; 3 Department of Pediatrics, Greifswald University Medical Center, 17475 Greifswald, Germany; jan.daebritz@med.uni-greifswald.de

**Keywords:** pediatric inflammatory bowel disease, patient empowerment, survey, parents, Crohn’s disease, ulcerative colitis, chronical ill children

## Abstract

Background: The number of children and adolescents with inflammatory bowel disease (IBD) is increasing. Many chronically ill children and adolescents have low health literacy. Patient empowerment (PE) enables positive changes and control over one’s disease through specific activities, information, and counseling. The CEDNA (IBD Needs Assessment) Survey aimed to provide the necessary data to improve PE in pediatric IBD (PIBD). Methods: Questionnaires were distributed to adolescent IBD patients and parents of children and adolescents with IBD throughout Germany. The answers were given anonymously. Based on the available data, a subgroup analysis was conducted in relation to the age of the patients and the period since diagnosis. For the parents’ responses, the same age groups were analyzed for comparison with the patients’ responses. Results: From October 2021 to April 2022, 2810 questionnaires were distributed and 1158 questionnaires were completed (*n* = 708 parents [61.1%], *n* = 450 patients [38.9%]). The results indicate that health literacy in children with IBD is low. Significant gaps in knowledge of important IBD topics were identified, and a comparison of responses regarding preferred methods and timing of obtaining information revealed differences between patient and parent preferences. The greatest need for knowledge on IBD topics was found in the group of 16–17-year-old patients on transition (*n* = 214, 31.8%) and in the group of patients diagnosed 1–2 years ago on the causes of IBD (*n* = 288, 17.4%). The willingness to seek advice was unexpectedly low. Conclusions: The analysis of all findings according to the patient’s age structure and duration since diagnosis can be used to enable age-appropriate communication at certain stages of the disease. This tailored information should increase patients’ health literacy, improve their management of the disease, and reduce the burden on their families.

## 1. Introduction

About a quarter of the 7 million people worldwide with inflammatory bowel disease (IBD) are diagnosed in childhood or adolescence [1]. The two most common forms of IBD are Crohn’s disease and ulcerative colitis. While incidence and prevalence for adult IBD appear to be stabilizing at high levels in western industrialized countries, they continue to rise in childhood and adolescence in many countries [2,3,4]. The prevalence of pediatric IBD (PIBD) in Germany was estimated to be 66.29 per 100,000 in 2012, which is higher than in most studies worldwide [5]. In contrast to adult onset of IBD, pediatric onset is often characterized by a more severe disease course, more extensive gastrointestinal involvement, and an increased need for care [6].

In addition to the challenges of growing up and personal development, children and adolescents with IBD must cope with the stress of chronic illness and disease management [7]. The personal transition to adulthood is further complicated by the medical transition from pediatric to adult health care during teenage years [8]. All developmental processes can be disrupted by symptoms of active disease or treatment difficulties that can lead to psychological comorbidities and social limitations in school and daily activities [9,10,11,12]. At the same time, healthcare utilization increases when patients have low health literacy [13]. Another consequence is a reduced quality of life and expanded health care use [9,14]. Health literacy describes a person’s ability to understand and process health-related information, especially to have more control over life events and situations [15]. Health literacy skills are the cognitive and social skills which determine the motivation and ability of individuals to gain access to information and understand and use it in ways which promote and maintain good health [16]. An important resilience factor for children and adolescents with chronic diseases is acceptance of the disease, which is promoted by support from their social environment such as their family and medical team [17]. PE is characterized by the accumulation of disease-related information and knowledge, as well as the involvement in activities such as participation and co-decision making that enable self-care [18]. PE aims to facilitate positive behavior change and increase patients’ health literacy. The goal is to increase control over one’s illness and to achieve an improved psychological status and optimized self-care [19]. PE (i.e., allowing people to have a say in decisions that affect their health) is an important tool, especially in chronic diseases that tend to impact people throughout their lives. When people have a say, they are more likely to comply with treatment plans and improve their problem-solving skills in relation to their own health [13,20,21]. To achieve empowerment, chronically ill children and adolescents, together with their parents and families, should be supported from the point of IBD diagnosis. In this way, they will know early on how to manage their condition well and acquire these skills for the rest of their lives [21].

In this article, we present a detailed subgroup analysis of the results of the first evaluation of the representative IBD Needs Assessment Survey (CEDNA) on the attitudes towards PIBD and the information needs of patients with IBD and parents. An overview of the study and overall results were published in 2023 [22]. The aim of the study was to investigate the self-management skills of adolescents and their families. Although clinicians have recognized the importance of PE in children and adolescents with chronic diseases, there are still no standardized approaches for assessment [20]. Our results show that patients have a low level of knowledge about their disease and still have a need to acquire health-related knowledge. To determine the extent of PE in children and adolescents with IBD, the information needs and coping activities of patients and parents were analyzed regarding age and duration since diagnosis for supporting age- and target group-specific information services and especially the development of future information strategies.

## 2. Methods

### 2.1. Data Basis

The data presented in this article are based on the CEDNA survey, which emerged from the CED-KQN (IBD Children’s and Adolescents’ Quality of Care Improvement Network) project. The project aimed to improve the care of children and adolescents with IBD by exploring various aspects of health services research in the context of eHealth and Big Data. CEDNA is the German acronym for representative IBD needs assessment survey.

A detailed description of the methodology of the CEDNA survey has been published previously [20]. In brief, for the CEDNA survey, two versions of a questionnaire were developed and distributed throughout Germany from October 2021 to April 2022: a questionnaire for adolescent patients with IBD aged 12–17 years (28 questions) and a questionnaire for parents of children and adolescents with IBD aged 0–17 years (41 questions), each accompanied by a patient information sheet (Supplementary File S1: Questionnaires for patients and parents). The parents were asked for the age of their children to be able to match parents’ answers with those of the age-group of the patients. The answers were given anonymously. The questionnaires could be completed either on paper or online. The questions were designed to identify the challenges of the disease for specific target groups. Socio-demographic data (e.g., age, gender, place of residence, family environment) were also collected to be able to interpret the anonymous data and to identify age-specific and duration of diagnosis specific differences.

Here, subgroup analyses of the data regarding age structure and duration since diagnosis were conducted to study the question of coping and information needs in more detail. Processing of the completed questionnaires took place at the study centre in Giessen, Germany. Hereby, the responses from the online questionnaires were imported from the LimeSurvey tool (LimeSurvey GmbH, Hamburg, Germany) into Excel (Office 365, Microsoft Corporation, Redmond, WA, USA), and the responses from the printed questionnaires were manually digitized into Excel. These two Excel lists were merged and analyzed using SAS statistical software (version 9.4) for descriptive evaluation and subgroup analyses.

### 2.2. Presentation of Results

The terms “patients”, “adolescents”, or “affected persons” are used to refer to the target group surveyed between the ages of 12 and 17. The term “parents” refers to all parents who completed the questionnaire for their sick children up to the age of 17.

For some questions, participants did not answer or did not answer with the given answer options (e.g., multiple answers were given even if only one option should have been answered). The missing answers and the incorrect answers were not included in the evaluations.

When presenting the subgroups, only those responses could be analyzed for which both the questions were answered and the age and duration since diagnosis were provided. 

The magnitude of these incorrect or missing answers was median = 0.15, P25 = 0.09 to P75 = 0.19 for the patient questionnaires and median = 0.22, P25 = 0.08 to P75 = 0.27 for the parent questionnaires.

## 3. Results

### 3.1. General Information about Patients and Their Disease

**Figure 1:**
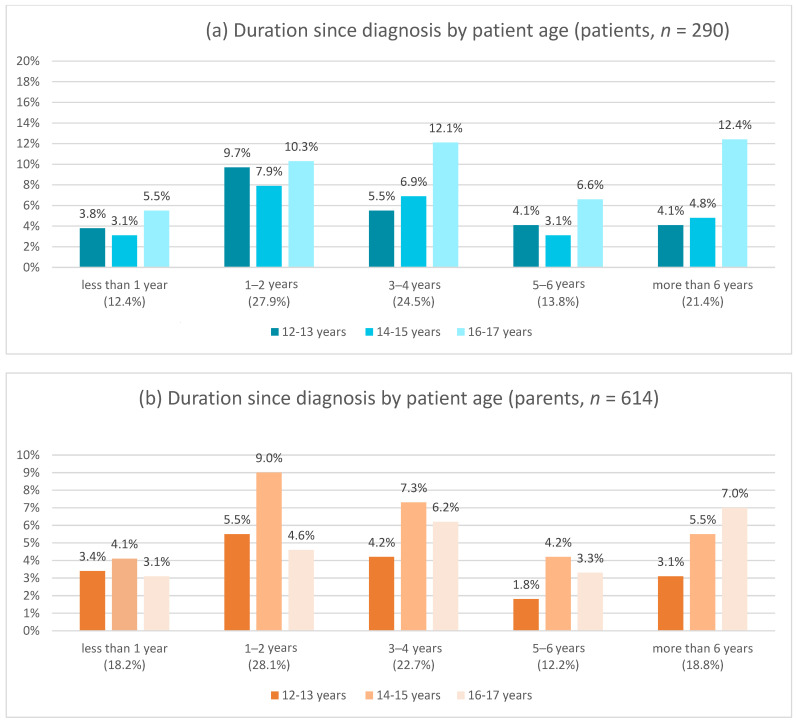
Duration of IBD among patients who participated in the survey (**a**) and according to parents who completed the questionnaire for their affected children (**b**). Breakdown of duration since diagnosis by age group between 12 and 17 years according to patients and parents, respectively.

The CEDNA survey was conducted from October 2021 to April 2022, during which 2810 questionnaires were distributed and 1158 questionnaires were completed (*n* = 708 parents [61.1%], *n* = 450 patients [38.9%]). An overview of all socio-demographic data as well as the type and course of the disease of the patients who participated in the CEDNA survey and of the parents who completed the survey for their children was previously published [22]. To understand how long children with IBD, or parents of children with IBD, have been dealing with the disease, an analysis was made with a breakdown of patients by age between 12 and 17 years and by duration since diagnosis of all types of IBD mentioned (Figure 1).

### 3.2. Main Contacts for Patients and Parents

As described previously, patients and parents were asked who their main healthcare provider for PIBD in the past 12 months was. For the vast majority, this was a pediatric gastroenterologist (patients 80.0%, *n* = 460; parents 96.6%, *n* = 210). The pediatrician was the main contact for 12.4% of patients (*n* = 460; parents 1.4%, *n* = 210) and the general practitioner was the main contact for 3.9% of patients (parents 0.5%, *n* = 210). An adult gastroenterologist was the primary contact for 2.6% of patients (parents 1.0%, *n* = 210). There was no primary contact for 1.1% of patients (*n* = 460; parents 0.5%, *n* = 210). Parents were also asked if a pediatric gastroenterologist was usually responsible for their child’s medical care, and 98.9% (*n* = 611) answered in the affirmative.

### 3.3. Activities to Cope with Disease

Patients indicated how often they had used a list of activities and services to cope with their IBD in the three months prior to the survey. They were given the option of answering yes or no. Parents were asked the same question for their children with IBD and were asked to rate whether the activity was never, rarely, regularly, or often done. Based on the available data, a subgroup analysis was performed according to the age of the patients. The responses were analyzed in groups (12–13 years, 14–15 years, and 16–17 years). Only questionnaires in which an age was specified were included. Parents’ answers were grouped according to the age groups of their children. The answers of the parents were then compared to the three age subgroups of patients accordingly (Table 1).

### 3.4. Information Needs of Patients and Parents on IBD Topics

**Figure 2:**
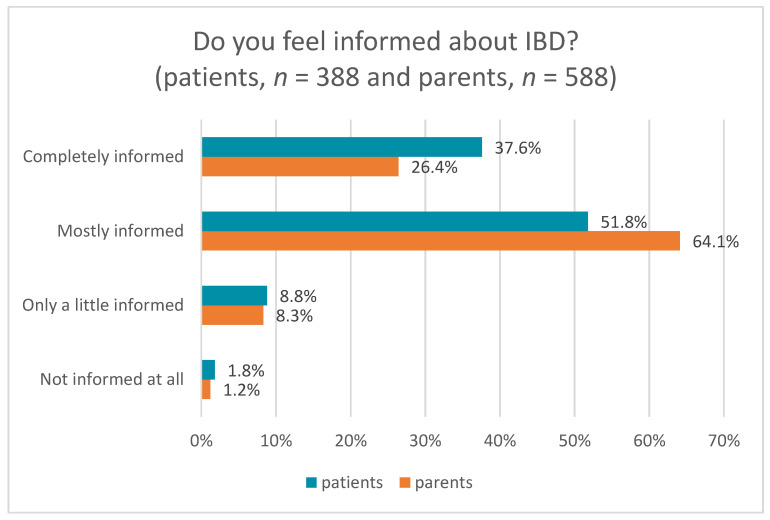
Level of how well-informed patients and parents feel about IBD in general.

To determine the level of knowledge on IBD topics, all patients and parents were asked whether they felt sufficiently informed and educated about IBD. They were given the choice of categorizing their response as “completely informed”, “mostly informed”, “only a little informed”, or “not informed at all” (Figure 2) [22].

Patients and parents were asked about their level of knowledge about IBD topics in general by filling in a scale from fully informed, mostly informed, and little informed to not informed at all. The responses of both groups were categorized according to the age of the patients and the time since diagnosis (Table 2). 

Both patients and parents were given a list of IBD-related topics to assess their information needs. Respondents were asked to indicate whether they would like more information on each of these topics. Most patients (60.7%, *n* = 290) wanted information about the causes of IBD (parents 22.0%, *n* = 322), followed by transition from pediatric into adult care (patients 58.5%, *n* = 284; parents 22.4%, *n* = 308), socio-legal issues (patients 52.6%, *n* = 274; parents 22.2%, *n* = 311), and IBD comorbidities (patients 51.4%, *n* = 284; parents 18.1%, *n* = 320). 

Looking at the information needs of patients in terms of age structure and duration since diagnosis, the group of patients with the greatest need for knowledge was those aged 16–17 years, followed by those aged 14–15 years, and then those aged 12–13 years. In terms of duration since diagnosis, the group who had been diagnosed 1–2 years ago wanted to know the most about IBD issues, followed by those who had been diagnosed 3–4 years ago, and then those who had been diagnosed more than 6 years ago.

For all topics, the responses from patients and parents regarding information needs on IBD topics are broken down by duration since diagnosis and age (12–17 years) of the patient (Table 3).

### 3.5. Preferred Sources of Information

Patients and parents were asked about their preferred sources of information on IBD topics. They were able to choose from a variety of digital, analog, and interactive formats. Responses were broken down by age (12–17 years for patients and 0–17 years for children of parents who completed the survey) to illustrate the differences between age groups (Table 4).

### 3.6. Provision of Information about IBD 

Patients and parents were asked to identify the appropriate professional, institution, or organization to contact for information about IBD. Pediatric and adult gastroenterologists were the two most important sources of information, followed by researchers. All responses regarding the provision of information about IBD were also presented by length of time since diagnosis (Table 5).

### 3.7. Preferred Timing of Relevant Information at Different Stages of the Illness

Parents have different information needs during the course of their children’s disease. They were given a list of different topics and asked to classify them according to the most appropriate time to provide information during the course of IBD. The possible classifications were topics at the duration of diagnosis, in the first year after diagnosis, or thereafter.

The most important topics at diagnosis were IBD in general (parents 67.6%, *n* = 552), causes (parents 63.2%, *n* = 535), and nutrition (parents 49.8%, *n* = 538). The overall data published previously [22] were analyzed in subgroups according to age. In the first year after diagnosis, parents rated school and vocational training (parents 28.2%, *n* = 528), vaccinations (parents 27.9%, *n* = 523), and disease documentation (parents 27.2%, *n* = 492) as relevant topics. As the disease progresses, parents prioritize the transition to adult health care (parents 84.2%, *n* = 525), sexuality (parents 70.5%, *n* = 484), and family planning (parents 69.7%, *n* = 475).

The responses of the parents as to when they feel that certain information about IBD topics is most useful in terms of the duration since diagnosis were analyzed (Table 6).

## 4. Discussion

The aim of the CEDNA survey was primarily to reflect on the current care situation of children and adolescents with IBD in Germany. Little is known about the additional support that is needed for patients and their families and how this need can be met. In a first step, the analysis of the overall results of the survey regarding disease, medical and psychological care, dealing with the disease, knowledge about the disease, and how to find information were published to create a database for the improvement process of communication between healthcare teams, patients, and other stakeholders as elements contributing to patient empowerment [22]. Limitation of the study were described elsewhere [20].

One of the findings from the initial evaluation of the CEDNA survey was the low health literacy of both patients and parents, so that in a second step here, the data were analyzed in more detail according to age and duration since diagnosis. The aim was to identify the characteristics of each subgroup, more precisely to determine the causes of their low health literacy. Therefore, the activities to cope with IBD, the information needs, the source of information and how information should be communicated as well as the timing of information as needed by children with IBD, and parents were identified.

The most important activities for patients to cope with the disease were having a hobby, talking to their doctor about the disease, and meeting friends, followed by talking about IBD with family and/or friends. Doing sport also plays an important role. If we look at the age-specific development of the activities that are important for children and adolescents with IBD, we see that there is a change between the ages of 12–13 and 16–17. The family plays an important role as a point of contact for 12–13-year-olds and becomes less important when patients reach the age of 16–17. However, it becomes more important to talk to the doctor about IBD as they get older. In a market research analysis among children and young people between 12 and 19 years in Germany conducted in 2023 [23], similar results were obtained. Pursuing hobbies (“doing sports”) was the second most common answer behind “being with friends” with 70.0% of responses. “Activities with the family” was the third most common interest with 30.0%. A comparison with the literature shows that our surveyed patients request similar leisure activities to their healthy peers. This should be considered in consultations with the patients to enable them to perform activities similar to those of healthy children.

The survey examined the information needs of the patients and the parents on several IBD topics. Just over one-third of the patients felt “completely” informed about IBD, compared to about one-quarter of the parents. Half of the patients and just over half of the parents felt “mostly” informed. This is important as a recent systematic literature review concluded that poor parental health literacy is associated with poorer child health outcomes [24]. To better address this association in the future counseling services, we conducted subgroup analyses by age and disease duration to identify differences between these groups. Based on the results of the present study, appropriate information services should be developed that take patients’ age and time since diagnosis into account. The level of knowledge in our survey is highest at the beginning of diagnosis, and after a period of more than 6 years, patients and parents should therefore receive more information, especially in the years after diagnosis, depending on individual needs. To the best of our knowledge, there are no information services that provide relevant content in such a timely manner. Closing knowledge gaps is a form of preventive measure so that negative consequences of illness can be recognized early and counteracted.

More than half of the patients wanted to know more about IBD causes, followed by transition to adult health care system and social issues. Patients with the greatest need for knowledge were 16–17 years old, followed by the 14–15 year-olds, and then the 12–13 year-olds. In terms of duration since diagnosis, the group who were diagnosed 1–2 years ago wanted to know the most about IBD issues, followed by those who were diagnosed 3–4 years ago. In personal interviews conducted in 2023 in Germany among 16 to 26-year-olds on the question of which topics are particularly important and desirable in their lives [25], 90% of interviewees stated “having good friends, close relationships with other people”, 80.3% “having a lot of fun, enjoying life”, and 71.4% “being there for the family, doing something for the family”. Regardless of the specific IBD topics, the comparison of the data shows that patients with IBD have the same wishes as healthy peers, extended by IBD topics to be able to achieve the goals mentioned. From this, we conclude that young people with IBD carry a greater burden on their way to reach the age of majority. Transition to adult health care and becoming an adult (social issues) play an important role and must be considered in counseling services. Transition has many challenging aspects [26]. The topic of transition medicine should therefore be given more attention and implementation in the future.

There is a clear difference in the media use of patients and parents when it comes to obtaining information about the disease. While patients prefer digital media, parents prefer print media and information via the internet. This corresponds to the general media development in Germany from 2004 to 2023. The use of print media by young people is declining [23], and the duration of daily online use by 12 to 19-year-olds in Germany increased from 99 min per day in 2006 to 224 min in 2023 [23]. An analysis of the age distribution of patients according to their preferred media for accessing information shows that all age groups prefer digital devices and over 95% of young people between the ages of 12 and 19 in Germany own a smartphone [23]. The trend towards digitalization should also be considered in patient advice by providing more digital services for children and young people.

For both patients and parents, pediatric gastroenterologists were the most important source of information about IBD, followed by the adult gastroenterologist for patients and nutritionists for parents. The third most important source for both patients and parents were active researchers. Trust in people and institutions can be an important aspect in providing helpful information. Regarding the duration since diagnosis, it is noticeable that the sources of information mentioned were most frequently used in the first two years after the diagnosis. This is the time after the onset of the disease when gathering general information becomes less important and the disease has a greater impact on daily life [27]. As part of a Germany-wide survey conducted by Bielefeld University between February and June 2022, 751 young people between the ages of 12 and 16 were asked about their trust in the media and public institutions [28]. Trust in public institutions ranged from around 55% to around 80%, and 76% of respondents said they trusted scientists, while only 24% said they had no trust in scientists.

Questions in our survey also referred to the appropriate times to receive information. During the disease, patients and parents had different wishes and needs for receiving information, depending on the disease duration since diagnosis. The most important topics at the duration since diagnosis were those related to IBD in general, IBD causes, and diet. In the first year after diagnosis, parents rated school and education, vaccinations, and disease documentation as relevant topics. During the further course of the disease, parents prioritized topics such as the transition to adult health care. In a study of the impact on symptom management in 838 Chinese and US patients, the authors concluded that the dynamics of health information processing need to be recognized and a more nuanced approach to patient support and care needs to be adopted [29]. We also conclude from the available data that providing the right information at the right time is an important part of successful future counseling.

## 5. Conclusions

Our overall findings from the CEDNA survey highlight and describe how parents and patients deal with information needs, transfer and timing of information, and coping with the activities they experience in relation to their IBD. This can be used in future counseling services to achieve positive effects on health-related quality of life and increased resilience. The detailed analysis presented here according to patient age and IBD duration can be used to provide age-appropriate communication at defined stages of the disease. Tailored information is intended to increase patients’ health literacy, improve their management of the disease, and reduce the burden on their families.

## Figures and Tables

**Table 1:** Activities to cope with IBD as reported by patients who completed the CEDNA survey themselves and by parents who completed the CEDNA survey for their children with the disease. The responses are grouped according to the age of the patients. Patient data shown in turquoise. Parents’ data shown in orange.

Responses in %																				
	Patients							Parents												
**Activity**	***n***	**Yes**			**No**			***n***	**Never**			**Rarely**			**Regularly**			**Often**		
**Pursue hobbies**	380	**84.7**			**15.3**			**582**	**8.4**			**13.5**			**46.0**			**32.1**		
Age (in years)		12–13	14–15	16–17	12–13	14–15	16–17		12–13	14–15	16–17	12–13	14–15	16–17	12–13	14–15	16–17	12–13	14–15	16–17
	273	23.4	25.3	37.4	2.9	1.5	9.5	578	1.2	3.5	1.7	0.0	3.8	3.5	9.9	13.1	11.1	5.5	10.7	7.6
**Talk to the doctor about IBD**	372	**83.3**			**16.7**			**587**	**6.8**			**33.4**			**53.2**			**6.6**		
Age (in years)		12–13	14–15	16–17	12–13	14–15	16–17		12–13	14–15	16–17	12–13	14–15	16–17	12–13	14–15	16–17	12–13	14–15	16–17
	269	20.4	23.0	39.4	7.1	3.3	6.7	583	0.7	2.1	0.9	7.9	8.7	7.7	8.6	18.2	14.6	1.0	2.1	1.2
**Meet friends**	376	**83.2**			**16.8**			**583**	**7.5**			**19.9**			**43.4**			**29.2**		
Age (in years)		12–13	14–15	16–17	12–13	14–15	16–17		12–13	14–15	16–17	12–13	14–15	16–17	12–13	14–15	16–17	12–13	14–15	16–17
	273	22.7	22.0	37.7	3.3	4.8	9.2	579	1.4	1.6	1.9	2.9	7.3	4.5	10.0	13.6	8.5	4.1	8.5	8.6
**Talk about IBD with family/friends**	380	**82.6**			**17.4**			**595**	**7.6**			**39.3**			**39.2**			**13.9**		
Age (in years)		12–13	14–15	16–17	12–13	14–15	16–17		12–13	14–15	16–17	12–13	14–15	16–17	12–13	14–15	16–17	12–13	14–15	16–17
	273	24.9	20.5	17.0	3.7	4.4	9.5	591	0.5	2.5	1.7	8.0	12.2	9.6	7.1	11.2	11.0	2.4	4.9	2.0
**Do sports and be physically active**	370	**74.3**			**25.7**			**595**	**7.6**			**39.3**			**39.2**			**13.9**		
Age (in years)		12–13	14–15	16–17	12–13	14–15	16–17		12–13	14–15	16–17	12–13	14–15	16–17	12–13	14–15	16–17	12–13	14–15	16–17
	268	19.8	20.9	34.3	7.5	5.2	12.3	589	1.4	2.9	1.9	4.2	8.1	7.1	8.0	13.6	11.4	4.8	6.3	3.7
**Take it easy physically**	355	**47.6**			**52.4**			**580**	**18.1**			**38.6**			**31.2**			**12.1**		
Age (in years)		12–13	14–15	16–17	12–13	14–15	16–17		12–13	14–15	16–17	12–13	14–15	16–17	12–13	14–15	16–17	12–13	14–15	16–17
	258	12.4	12.0	23.3	14.3	13.6	24.4	576	1.9	6.4	4.2	9.0	9.7	9.7	5.6	9.7	7.3	1.6	5.0	3.1
**Pay particular attention to the diet**	354	**38.7**			**61.3**			**586**	**17.4**			**33.8**			**33.1**			**15.7**		
Age (in years)		12–13	14–15	16–17	12–13	14–15	16–17		12–13	14–15	16–17	12–13	14–15	16–17	12–13	14–15	16–17	12–13	14–15	16–17
	258	8.9	9.7	19.0	19.0	16.7	26.7	582	4.1	4.1	4.6	6.7	12.0	7.6	5.5	11.0	7.9	1.7	4.0	4.0
**Talk about IBD with others affected**	358	**20.7**			**79.3**			**575**	**73.6**			**20.8**			**4.0**			**1.6**		
Age (in years)		12–13	14–15	16–17	12–13	14–15	16–17		12–13	14–15	16–17	12–13	14–15	16–17	12–13	14–15	16–17	12–13	14–15	16–17
	260	6.2	5.0	11.2	21.9	20.8	35.0	571	12.1	21.7	18.4	4.4	7.5	4.9	0.4	1.4	1.4	0.4	0.4	0.2
**Perform relaxation exercises**	356	**19.1**			**80.9**			**567**	**65.8**			**27.5**			**4.8**			**1.9**		
Age (in years)		12–13	14–15	16–17	12–13	14–15	16–17		12–13	14–15	16–17	12–13	14–15	16–17	12–13	14–15	16–17	12–13	14–15	16–17
	258	4.3	6.2	10.9	23.3	19.8	35.7	564	12.9	20.7	14.5	4.8	7.6	6.7	0.7	1.8	1.8	0.0	0.9	0.7
**Use psychological counseling**	357	**16.0**			**84.0**			**576**	**77.8**			**10.0**			**10.6**			**1.6**		
Age (in years)		12–13	14–15	16–17	12–13	14–15	16–17		12–13	14–15	16–17	12–13	14–15	16–17	12–13	14–15	16–17	12–13	14–15	16–17
	260	24.6	21.5	35.4	3.5	4.6	10.4	572	14.2	23.8	17.7	1.9	3.7	2.6	2.3	3.1	3.1	0.2	0.0	0.7
**Take part in a self-help group**	359	**0.8**			**99.2**			**579**	**97.4**			**1.7**			**0.7**			**0.2**		
Age (in years)		12–13	14–15	16–17	12–13	14–15	16–17		12–13	14–15	16–17	12–13	14–15	16–17	12–13	14–15	16–17	12–13	14–15	16–17
	258	0.0	0.0	0.8	27.5	26.4	45.3	575	18.4	29.9	22.8	0.2	0.3	1.0	0.0	0.2	0.3	0.0	0.2	0.0

**Table 2:** Level of knowledge as reported by patients and parents. The patients and the parents filled in a scale from fully informed, mostly informed, and little informed to not at all informed. The answers were divided according to the age of the patients and duration since diagnosis. Patient data shown in turquoise. Parents’ data shown in orange.

Responses in %								
**Patients (*n* = 289)**								
**Knowledge**	**Age**			**Duration since Diagnosis**				
	**12–13**	**14–15**	**16–17**	**<1 Year**	**1–2 Years**	**3–4 Years**	**5–6 Years**	**>6 Years**
Fully informed	41.8	37.3	35.8	46.0	32.4	37.9	41.5	35.7
Mostly informed	43.0	54.7	52.6	40.0	56.5	47.4	50.9	53.6
Little informed	15.2	6.7	8.8	12.0	6.5	12.6	5.7	7.1
Not enough informed	0.0	0.0	2.2	2.0	0.9	2.1	1.9	2.4
**Parents (*n* = 586)**								
**Knowledge**	**Age**			**Duration since diagnosis**				
	**12–13**	**14–15**	**16–17**	**<1 Year**	**1–2 Years**	**3–4 Years**	**5–6 Years**	**>6 Years**
Fully informed	28.6	34.5	25.5	19.2	23.4	33.1	28.8	28.6
Mostly informed	44.7	59.8	66.7	67.3	68.3	55.4	65.8	63.4
Little informed	7.6	5.8	7.8	11.5	6.6	10.8	4.1	8.0
Not enough informed	0.0	1.2	0.0	1.9	1.8	0.8	1.4	0.0

**Table 3:** Information needs of patients (aged 12–17 years) and parents. Presentation of all relevant topics related to IBD and survey participants’ assessment of whether they would like to know more about these topics, broken down by age and duration since diagnosis. Patient data shown in turquoise. Parents’ data shown in orange.

Responses (Yes, I Would Like to Know More about These Topics) in %					
Topics	Patients	Parents		Patients	Parents
**Causes**	**60.7**	**22.0**	**Drug Treatment Options**	**44.0**	**20.8**
Disease duration	*n* = 288	*n* = 321	Disease duration	*n* = 282	*n* = 331
<1 year	8.7	4.7	<1 year	5.0	3.9
1–2 years	17.4	8.1	1–2 years	13.5	8.8
3–4 years	15.6	5.3	3–4 years	11.3	4.2
5–6 years	8.0	1.6	5–6 years	6.4	1.2
>6 years	10.8	2.5	>6 years	7.8	2.7
Age	*n* = 211	*n* = 321	Age	*n* = 212	*n* = 331
12–13 years	9.6	3.7	12–13 years	10.4	3.9
14–15 years	23.9	5.6	14–15 years	11.3	5.4
16–17 years	20.3	6.5	16–17 years	25.0	4.5
**Transition**	**58.5**	**22.4**	**Nutrition**	**41.3**	**19.7**
Disease duration	*n* = 283	*n* = 307	Disease duration	*n* = 274	*n* = 319
<1 year	6.7	2.9	<1 year	6.2	3.1
1–2 years	17.3	6.2	1–2 years	12.4	6.9
3–4 years	13.4	5.9	3–4 years	8.8	5.0
5–6 years	8.1	3.6	5–6 years	5.8	1.9
>6 years	13.1	3.9	>6 years	7.7	2.8
Age	*n* = 214	*n* = 307	Age	*n* = 208	*n* = 319
12–13 years	12.1	4.9	12–13 years	7.7	3.4
14–15 years	16.8	7.5	14–15 years	9.6	4.7
16–17 years	31.8	6.8	16–17 years	25.0	4.4
**Concomitant diseases**	**51.4**	**18.1**	**Preventive health care**	**42.1**	**17.9**
Disease duration	*n* = 282	*n* = 319	Disease duration	*n* = 270	*n* = 311
<1 year	7.4	3.8	<1 year	7.8	2.6
1–2 years	15.2	5.0	1–2 years	10.4	5.8
3–4 years	13.1	4.7	3–4 years	11.1	4.8
5–6 years	7.1	2.5	5–6 years	4.8	1.6
>6 years	8.2	2.2	>6 years	8.1	3.2
Age	*n* = 211	*n* = 319	Age	*n* = 203	*n* = 311
12–13 years	11.4	3.1	12–13 years	8.9	3.2
14–15 years	14.7	5.3	14–15 years	8.9	4.5
16–17 years	27.0	5.3	16–17 years	24.1	4.8
**Social and law issues**	**52.6**	**22.2**	**Medication side effects**	**38.0**	**17.6**
Disease duration	*n* = 272	*n* = 310	Disease duration	*n* = 282	*n* = 323
<1 year	7.7	3.5	<1 year	4.6	2.5
1–2 years	14.7	6.5	1–2 years	9.9	6.2
3–4 years	11.0	4.5	3–4 years	10.3	5.0
5–6 years	8.5	3.9	5–6 years	6.4	1.5
> 6 years	10.7	3.9	>6 years	6.7	2.5
Age	*n* = 205	*n* = 310	Age	*n* = 212	*n* = 321
12–13 years	10.2	4.5	12–13 years	9.4	2.8
14–15 years	12.2	6.1	14–15 years	8.0	5.9
16–17 years	31.7	5.5	16–17 years	22.2	5.0
**Complications in the course of the disease**	**49.5**	**17.0**	**Patient Organizations**	**17.8**	**10.7**
Disease duration	*n* = 281	*n* = 317	Disease duration	*n* = 268	*n* = 298
<1 year	7.1	2.8	<1 year	1.1	2.3
1–2 years	13.9	5.4	1–2 years	3.4	3.7
3–4 years	13.2	5.4	3–4 years	6.3	1.3
5–6 years	6.4	1.6	5–6 years	3.0	1.3
>6 years	8.9	1.9	>6 years	3.7	2.0
Age	*n* = 210	*n* = 317	Age	*n* = 203	*n* = 298
12–13 years	14.3	3.2	12–13 years	3.4	2.0
14–15 years	13.8	5.0	14–15 years	4.9	2.4
16–17 years	23.8	5.4	16–17 years	11.3	2.0
**School and training**	**47.3**	**20.3**	**Self-help groups**	**17.8**	**9.2**
Disease duration	*n* = 278	*n* = 314	Disease duration	*n* = 250	*n* = 393
<1 year	6.5	3.2	<1 year	2.0	3.0
1–2 years	14.7	6.7	1–2 years	4.4	3.3
3–4 years	12.2	4.5	3–4 years	4.4	1.0
5–6 years	5.8	2.9	5–6 years	2.0	1.0
>6 years	7.6	3.2	>6 years	4.0	1.0
Age	*n* = 213	*n* = 314	Age	*n* = 118	*n* = 302
12–13 years	12.2	3.2	12–13 years	2.7	0.9
14–15 years	12.7	3.5	14–15 years	6.4	2.3
16–17 years	25.4	5.1	16–17 years	9.6	1.3
**Dealing with mental strain and stress**	**46.6**	**20.2**	**IBD in general**	**42.9**	**15.7**
Disease duration	*n* = 279	*n* = 321	Disease duration	*n* = 273	*n* = 331
<1 year	4.7	3.1	<1 year	6.6	3.9
1–2 years	12.2	6.5	1–2 years	11.0	6.9
3–4 years	11.8	5.6	3–4 years	12.1	3.3
5–6 years	7.5	1.9	5–6 years	5.5	0.9
>6 years	10.0	3.1	>6 years	6.6	0.6
Age	*n* = 214	*n* = 321	Age	*n* = 205	*n* = 331
12–13 years	9.8	4.4	12–13 years	8.8	1.5
14–15 years	12.1	4.7	14–15 years	12.7	3.9
16–17 years	26.2	5.0	16–17 years	25.4	4.2
**Surgical treatment options**	**31.5**	**11.0**	**Prognosis**	**43.8**	**17.2**
Disease duration	*n* = 288	*n* = 316	Disease duration	*n* = 270	*n* = 313
<1 year	2.8	2.8	<1 year	6.3	3.8
1–2 years	9.7	3.8	1–2 years	11.5	5.8
3–4 years	8.7	2.8	3–4 years	11.5	3.2
5–6 years	5.2	0.9	5–6 years	5.2	1.6
>6 years	4.9	0.6	>6 years	9.3	2.9
Age	*n* = 216	*n* = 316	Age	*n* = 204	*n* = 313
12–13 years	7.9	0.9	12–13 years	10.8	2.2
14–15 years	9.3	2.5	14–15 years	12.3	5.1
16–17 years	14.8	3.2	16–17 years	23.0	4.8

**Table 4:** Method of preferred information gathering on IBD topics as reported by patients and parents. Patient data shown in turquoise. Parents’ data shown in orange.

Responses (in This Way I Would Like to Have Information) in %												
**Source of Information**	***n***	**Patients**	***n***	**Parents**	**Source of Information**				***n***	**Patients**	***n***	**Parents**
Internet	375	57.9	578	60.6	Specialist magazines and books				376	33.2	578	48.8
YouTube	375	54.7	576	22.9	Brochures and information leaflets				376	32.4	578	44.8
Explanatory films	375	47.2	577	40.7	Forums for patients/parents				375	32.0	577	50.8
Lectures	375	42.7	578	54.0	Apps (communication platform)				375	28.3	578	18.3
Books for children/adolescents	376	37.5	578	57.4	Blogs				375	25.3	577	10.7
Workshops (1 day)	376	35.4	578	47.6	Chats				375	21.1	577	9.5
Apps (online information)	374	35.3	576	27.3	Counseling offers for patients				375	17.3	577	41.1
**Preferred Information Gathering by Age** **Responses (in This Way I Would Like Information) in %**												
**Source of Information**	**Patients: Age in Years**				**Parents: Age of Their Children (Patients) in Years**							
	***n***	**12–13**	**14–15**	**16–17**	***n***	**0–5**	**6–7**	**8–9**	**10–11**	**12–13**	**14–15**	**16–17**
Internet	281	12.5	13.9	29.9	575	3.3	1.4	3.5	8.3	11.5	19.3	13.2
YouTube	281	12.5	15.3	29.5	573	1.0	0.5	1.7	4.4	3.8	6.1	5.4
Explanatory films	281	11.4	13.2	23.8	574	2.6	1.4	3.5	7.0	7.1	11.1	8.2
Lectures	281	12.1	11.4	19.6	575	3.0	1.2	4.2	7.3	9.7	16.3	12.5
Books for children/adolescents	282	11.3	11.3	16.7	575	4.7	2.4	4.5	10.4	11.5	15.5	8.7
Workshops (1 day)	282	8.5	10.3	17.7	575	3.0	1.2	3.1	7.3	8.7	14.6	9.7
Apps (online information)	280	7.1	10.4	18.2	573	2.6	1.0	2.3	3.7	4.0	8.4	5.1
Specialist magazines and books	282	7.8	7.4	18.1	575	2.3	1.6	2.8	6.6	9.0	14.8	12.0
Brochures and information leaflets	282	6.0	9.2	16.3	575	2.1	1.4	2.8	5.4	8.3	13.9	11.1
Forum for patients/parents	291	4.3	12.1	16.7	574	3.7	1.9	3.5	8.2	9.2	14.6	9.6
Apps (communication platform)	281	7.8	6.8	13.9	575	1.7	0.7	1.4	3.3	2.3	5.7	3.1
Blogs	281	3.2	6.8	14.9	574	0.2	0.5	0.5	2.3	1.7	3.5	1.9
Chats	291	4.6	6.0	10.0	574	0.9	0.5	0.9	1.6	1.2	3.0	1.4
Counseling offers for patients	281	2.8	5.7	8.9	574	2.8	1.6	1.7	5.2	7.8	10.3	11.3

**Table 5:** Who should provide information on IBD issues as indicated by patients (12–17 years) and by parents of patients (0–17 years) broken down by disease duration. Patient data shown in turquoise. Parents’ data shown in orange.

**Responses in %**													
**Information Source**	***n***	**Patients**	***n***	**Parents**		**Information Source**			***n***	**Patients**		***n***	**Parents**
Pediatric gastroenterologists	387	87.3	577	95.3		Sports Professionals			387	14.7		577	14.2
Adult gastroenterologists	386	56.2	576	40.6		Patient Associations			387	12.7		577	22.2
Active research scientists	387	40.8	577	43.2		Representatives of self-help groups			387	7.2		577	18.4
Nutritionists	387	37.7	577	52.9		Social Worker			387	5.7		577	9.0
Young people affected	387	33.9	577	38.5		Lawyers			387	3.4		577	6.4
Psychologists	387	25.8	577	38.8		Health insurance companies			387	3.1		577	14.0
Families affected	387	23.0	577	39.7		Outdoor and music educators			387	2.1		577	6.8
**Breakdown of Preferred Information Source by Duration Since Diagnosis** **Responses in %**													
	**Patients: Disease Duration in Years**							**Parents: Disease Duration of Child (Patients) in Years**					
**Information Source**	***n***	**<1**	**1–2**	**3–4**	**5–6**		**>6**	***n***	**<1**	**1–2**	**3–4**	**5–6**	**>6**
Pediatric gastroenterologists	384	11.5	25.5	20.1	11.2		19.3	575	17.0	27.3	20.7	12.2	18.1
Adult gastroenterologists	383	5.5	13.6	13.6	9.7		13.8	574	8.0	10.8	8.4	5.2	8.0
Active research scientist	384	5.5	12.8	8.9	5.5		8.6	575	8.2	12.7	8.9	4.7	8.5
Nutritionists	384	6.0	10.9	8.9	6.0		5.7	575	9.7	16.0	12.3	6.8	8.2
Young people concerned	384	3.9	9.4	7.3	6.0		7.0	575	7.1	12.9	8.0	4.7	5.9
Psychologists	384	3.9	5.2	6.0	5.2		5.5	575	6.4	10.3	9.4	5.2	7.5
Families affected	384	3.4	5.2	6.5	4.4		3.6	575	8.0	11.1	9.7	4.3	6.6
Sports professionals	384	2.3	3.6	3.4	1.8		3.1	575	1.7	4.2	4.0	1.7	2.6
Patient associations	384	1.3	3.9	2.9	2.1		2.6	575	3.3	7.1	4.9	3.3	3.7
Representatives of self-help groups	384	0.8	3.1	0.8	1.0		1.6	575	3.7	6.1	3.8	2.1	2.8
Social worker	384	0.3	2.1	1.0	0.3		2.1	575	1.6	3.1	2.3	1.2	0.9
Lawyers	384	0.5	0.5	0.5	0.3		0.8	575	0.7	1.7	1.6	1.2	1.2
Health insurance companies	384	0.3	1.3	0.5	0.3		0.8	575	2.1	5.0	2.4	2.3	2.3
Outdoor and music educators	384	0.0	0.8	0.8	0.0		0.5	575	0.7	2.3	2.4	0.9	0.5

**Table 6:** Preferred timing of information from parents (0–17 years). Presentation of all relevant topics related to IBD and assessment of the appropriate timing of information requested by parents. Parents’ data shown in orange.

At What Point Information Is Needed	*n*	At Diagnosis			In the First Year			During the Further Disease Course			No Need for Information		
IBD Topics								Responses in % and Age in Years					
General	552	67.6			10.5			17.0			4.9		
Age		12–13	14–15	16–17	12–13	14–15	16–17	12–13	14–15	16–17	12–13	14–15	16–17
	387	14.2	25.8	22.5	4.4	7.0	5.4	4.1	7.5	4.4	1.8	1.6	1.3
Causes	535	63.2			16.3			15.9			4.7		
Age		12–13	14–15	16–17	12–13	14–15	16–17	12–13	14–15	16–17	12–13	14–15	16–17
	372	14.0	25.5	22.6	4.3	7.0	5.6	4.3	7.5	4.3	1.9	1.6	1.3
Nutrition	538	49.8			22.1			21.9			6.1		
Age		12–13	14–15	16–17	12–13	14–15	16–17	12–13	14–15	16–17	12–13	14–15	16–17
	387	10.9	20.2	16.5	4.1	9.3	7.8	7.2	9.6	7.8	1.6	3.6	1.6
Medical treatment options	555	47.7			16.8			32.1			3.4		
Age		12–13	14–15	16–17	12–13	14–15	16–17	12–13	14–15	16–17	12–13	14–15	16–17
	400	10.8	18.0	17.8	3.3	8.0	5.0	9.8	15.8	8.8	0.8	1.3	1.0
Medication side effects	542	45.2			21.2			28.4			5.2		
Age		12–13	14–15	16–17	12–13	14–15	16–17	12–13	14–15	16–17	12–13	14–15	16–17
	389	10.0	18.3	16.2	4.4	9.0	8.2	8.0	13.1	8.5	2.3	1.5	0.5
Prognosis	536	37.3			20.5			36.6			5.6		
Age		12–13	14–15	16–17	12–13	14–15	16–17	12–13	14–15	16–17	12–13	14–15	16–17
	392	8.4	12.2	12.2	3.8	9.9	6.9	9.4	15.6	12.2	2.0	3.3	3.8
Dealing with psychological stress/stress management	532	33.1			26.1			32.7			8.1		
Age		12–13	14–15	16–17	12–13	14–15	16–17	12–13	14–15	16–17	12–13	14–15	16–17
	381	5.8	15.0	11.3	6.3	9.2	10.5	9.4	14.7	10.0	1.8	4.2	1.8
Psychotherapeutic accompanying measures	526	31.6			22.6			33.8			12.0		
Age		12–13	14–15	16–17	12–13	14–15	16–17	12–13	14–15	16–17	12–13	14–15	16–17
	375	5.1	12.3	12.3	5.3	9.6	8.0	9.9	14.7	10.4	3.7	5.6	3.2
Concomitant diseases	540	29.1			25.4			41.7			3.9		
Age		12–13	14–15	16–17	12–13	14–15	16–17	12–13	14–15	16–17	12–13	14–15	16–17
	387	7.8	10.6	11.4	3.9	10.9	9.6	11.6	18.9	11.9	1.0	2.1	0.5
School and vocational training	528	24.2			28.2			40.3			7.2		
Age		12–13	14–15	16–17	12–13	14–15	16–17	12–13	14–15	16–17	12–13	14–15	16–17
	384	7.6	10.4	7.3	6.3	12.8	10.2	9.6	16.4	2.1	2.1	3.6	
Patient organizations and self-help groups	489	25.8			24.1			31.7			18.4		
Age		12–13	14–15	16–17	12–13	14–15	16–17	12–13	14–15	16–17	12–13	14–15	16–17
	347	4.6	11.2	7.2	4.9	9.8	9.8	9.8	12.7	9.5	5.2	7.8	7.5
Measures for preventive health care	509	21.4			23.6			45.0			10.0		
Age		12–13	14–15	16–17	12–13	14–15	16–17	12–13	14–15	16–17	12–13	14–15	16–17
	368	4.1	8.7	6.8	4.6	9.2	8.4	13.0	19.8	15.8	2.2	5.2	2.2
Complications during course of the disease	532	19.7			19.4			56.0			4.9		
Age		12–13	14–15	16–17	12–13	14–15	16–17	12–13	14–15	16–17	12–13	14–15	16–17
	384	5.2	6.5	7.3	3.6	7.0	8.1	14.8	25.8	17.4	1.0	2.3	0.8
Social law issues	509	11.0			16.7			63.5			8.8		
Age		12–13	14–15	16–17	12–13	14–15	16–17	12–13	14–15	16–17	12–13	14–15	16–17
	369	2.2	5.1	3.5	3.5	6.8	4.9	17.6	27.6	20.9	1.4	3.0	3.5
Transition to adult health care	525	3.6			4.4			84.2			7.8		
Age		12–13	14–15	16–17	12–13	14–15	16–17	12–13	14–15	16–17	12–13	14–15	16–17
	383	1.0	1.6	0.8	0.5	1.3	3.1	21.4	37.9	27.9	1.0	1.8	1.6

## Data Availability

Restrictions apply to the availability of these data. The data were obtained from the quality improvement project CED-KQN (Big Data eHealth—Improving the Care of Children and Adolescents with IBD) which was funded by the Federal Joint Innovation Committee G-BA and will be available on the G-BA’s URL https://innovationsfonds.g-ba.de/projekte/versorgungsforschung/ced-kqn-big-data-ehealth-verbesserung-der-versorgung-von-kindern-und-jugendlichen-mit-chronisch-entzuendlichen-darmerkrankungen.171 from February 2024.

## Supplementary Materials

The following supporting information can be downloaded at: https://www.mdpi.com/article/10.3390/children11040481/s1, S1: Questionnaires for patients and parents.
