# Supplementary material for: Health Literacy of Children and Adolescents with Inflammatory Bowel Disease (IBD) and Parents of IBD Patients—Coping and Information Needs
Source: Children (Basel). 2024 Apr 17;11(4):481. doi: 10.3390/children11040481 (PMC11048777; doi:10.3390/children11040481)
Supplement: Supplementary file 1 [file children-11-00481-s001.zip › children-2942787-supplementary.pdf]

# CEDNA - The survey

as part of a study to improve the care of children and adolescents with inflammatory bowel disease (IBD).

VERSION IBD PATIENTS - for children and adolescents with IBD from 12 to 17 years

For **ease of reading,** the masculine, feminine, and various forms of the language are used interchangeably. All personal terms apply equally to all genders.

This survey is only for children and adolescents with inflammatory bowel disease (IBD). We ask that you complete the questionnaire without assistence if you are between the ages of 12 and 17 and have IBD.

You can also **complete** the **CEDNA online**. See the information on the last page.

First, we will ask you to enter your own **questionnaire code,** because the survey is **anonymous**, which means that no one can can find out that you have filled it out. With the help of the code it is possible to filter out duplicate questionnaires in the scientific analysis.

Please enter your **code** here following the instructions:

First letter of first name of your mother

Last letter of your first name

First letter of your place of residence

First two digits of your birthday (e.g. 4=04; 15=15)

# Information about your disease

**01. What IBD has your doctor diagnosed?**

- Crohn's disease
- Ulcerative colitis
- Unclassified IBD

**02. What concomitant diseases have you been diagnosed with?**
(Please check more than one box if applicable)

- Primary Sclerosing Cholangitis (PSC)
- Skin diseases (erythema nodosum, pyoderma gangraenosum)
- Eye diseases (e.g. uveitis, iridocyclitis)
- Gall and kidney stones
- Thrombosis
- Inflammatory joint disease (e.g. rheumatism, idiopathic arthritis)
- Psychological disorders (e.g. depression, anxiety disorder)
- Other:
- None
- I do not know

**03. How long have you been diagnosed with IBD?**

- Less than 1 year
- 1 - 2 years
- 3 - 4 years
- 5 - 6 years
- Longer than 6 years

**04. In the last 12 months, who was your main source of medical care for your IBD?** (Main contact person)

- No main contact
- Primary care physician
- Pediatrician
- Pediatric gastroenterologist
- Adult gastroenterologist
- Other, specifically:

**05. The course of IBD is usually intermittent. Phases of no symptoms or with minor symptoms (resting phase, remission) alternate with phases of severe symptoms (acute relapses). Which phase of the disease are you currently in?**

- Diagnostic phase
- Resting phase/Remission
- Relapse phase
- I do not know

# Medical and psychological care

**06. If you have a physical problem related to your IBD, can you contact someone who can give you the support you need?**

- Yes, always ➞ Go to question 07
- Now and then ➞ Go to question 07
- Rather not ➞ Go to question 08

**07. Who do you see if you have a physical problem related to your IBD?** (Please check more than one box if necessary).

- Doctor
- Parents and family
- Friends
- Other, name:

**08. If you have a mental or emotional problem related to your IBD, can you contact someone who can give you the support you need?**

- Yes, always ➞ Go to question 09
- Now and then ➞ Go to question 09
- Rather not ➞ Go to question 10

**09. Who can you turn to if you have a mental or emotional problem related to your IBD?** (Please check more than onebox if necessary).

- Doctor
- Parents and family
- Friends
- Other, name:

**10. Overall, are you satisfied with the medical care you receive for your IBD?**

- Yes
- No

# Managing chronic inflammatory bowel disease

**11. You probably use different ways to manage your IBD. Which of the following methods do you use?** (Think about the past three months)

| | **Yes** | **No** |
|---|---|---|
| Talking about IBD with other people who have it | | |
| Talking about IBD to family and/or friends | | |
| Talking about IBD to your doctor | | |

| |
|---|
| Getting together with friends |
| Participating in hobbies |
| Doing relaxation exercises (yoga, autogenic training, etc.) |
| Taking care of yourself physically |
| Exercise and be physically active |
| Pay special attention to your diet |
| Get psychological support |
| Participate in support group activities |
| Other, specifically: |

**12. How do you feel when you think about your IBD?**

| | Yes | No |
|---|---|---|
| I am afraid | | |
| I am ashamed | | |
| I am insecure | | |
| I am nervous | | |
| I am sad | | |
| I am calm | | |
| I am helpless | | |
| I am exhausted | | |
| I have no more courage | | |
| I feel lonely | | |
| I feel overwhelmed | | |
| I feel abandoned | | |
| I think everything will be okay | | |

**13. Have you been able to deal with your feelings about your IBD in the last three months?**

- Not at all
- Only a little
- Most of the time
- Totally

## How to get / share information

**The following questions are designed to help us understand, how and where you get information about IBD. What are your ideas and wishes for finding and sharing information about IBD?**

**14. Do you feel adequately educated and informed?**

- Not at all
- Only a little
- Mostly
- Totally

**15. There is a variety of topics related to CED. On which topic do you feel well informed and where would you like to know more?**

| | I feel good informed about it | | I would like to know even more about it | |
|---|---|---|---|---|
| | Yes | No | Yes | No |
| IBD in general | | | | |
| Causes | | | | |
| Drug treatment options | | | | |
| Surgical treatment options | | | | |
| Side effects of medications | | | | |
| Complications in the progressive course of the disease | | | | |
| Concomitant diseases | | | | |
| Prognosis | | | | |
| Preventive health measures (e.g. cancer screening, smoking cessation) | | | | |
| Complementary medicine (e.g. homeopathy) | | | | |
| Vaccinations | | | | |
| Nutrition | | | | |
| Coping with psychological stress and stress management | | | | |
| Psychotherapy | | | | |
| Transition to adult medicine (transition) | | | | |
| Sexuality issues | | | | |
| Social legal issues (employment promotion, health insurance, rehabilitation, disability law, pension insurance) | | | | |
| School and education (e. g. compensation for disadvantages, possibility to go to the toilet at any time, information at school) | | | | |
| Patient organizations & self-help groups | | | | |
| Travel abroad with IBD | | | | |
| Other, specifically: | | | | |

**16. IBD can present many challenges. Therefore, non-physician professionals and facilities are also involved in the care. Which of the following treatment or counseling services do you use or have used?** (Please check more than one box if applicable)

- o Nutrition counseling
- o Genetic counseling
- o Family counseling
- o Sexual counseling
- o Physical therapy
- o Occupational therapy
- o Psychological help/support
- o Stress management services (e.g. relaxation training, autogenic training)
- o Outpatient nursing services, home help
- o Support groups
- o Advice from health or long-term care insurance
- o Advice from pension insurance (e.g. rehab)
- o Events that provide information about the disease (e.g. attendance of a seminar)

- Transition programs (e.g. Berlin Transition Program)
- Other, specifically:
- I do not or have not received any treatment or counseling

**17. There are a variety of sources where you can find information about your IBD. Please indicate whether you find the following sources of information sources trustworthy and whether you have ever read about them.**

| | **I find this source of information about IBD trustworthy** | | **I have already read about the source of information on IBD** | |
|---|---|---|---|---|
| | YES | NO | YES | NO |
| Doctors | | | | |
| Pharmacists | | | | |
| Medical societies | | | | |
| Pharmaceutical industry | | | | |
| Health insurance companies | | | | |
| Non-medical practitioners | | | | |
| Nutritionists | | | | |
| Psychologists | | | | |
| Psychotherapists | | | | |
| Transition programs | | | | |
| Other patients | | | | |
| Patient associations (e.g. DCCV) | | | | |
| Support groups | | | | |
| Family, friends, peers | | | | |
| Specific Internet forums, chat rooms | | | | |
| Internet in general | | | | |
| Television, consumer programs | | | | |
| Scientific journals, textbooks | | | | |
| Politics | | | | |
| Other, specifically: | | | | |

**18. How would you like to receive information about IBD?** (Please check more than one box if applicable)

**Printed information media**

- Brochures and flyers
- Age-appropriate books for children and teens
- Professional journals and reference books
- Other, specifically:

**Information events**

- Lectures
- Conferences
- Other, specifically:

**Online information**

- Internet sites
- Patient counseling services
- Educational films
- Regular newsletters
- YouTube channel
- Blogs
- Apps
- Other, specifically:

**Online communication platforms**

- Forums for patients and/or parents
- Chats
- Apps
- Other, specifically:

**Informational seminars**

- One-day workshops
- Weekend seminar with overnight stay
- Weekend seminar without lodging
- Other, specifically:

**19. Who should give you the information about IBD?** (Please check all that apply)

- Adult specialists (gastroenterologists)
- Pediatric gastroenterologists
- Nurses
- Psychologists
- Current research scientists
- Nutritionists
- Sports specialists
- Social workers
- Experiential and music educators
- Patient associations
- Support group representatives
- Affected families
- Affected adolescents
- Lawyers
- Representatives of consumer protection/help centers
- Health insurance companies
- Others, specifically:

**20. Now we are talking about information sharing opportunities. Would you attend one of the following? How many people should participate?**

| | Yes | No | 2-5 | 6-10 | 11-15 | >15 |
|---|---|---|---|---|---|---|
| Group meeting of parents of affected children | | | | | | |
| Group meeting of affected children and teenagers | | | | | | |
| Group meetings of parents with their affected children | | | | | | |
| Family weekends for affected children, parents and siblings | | | | | | |
| Online communication platforms (e.g. forums, chats) | | | | | | |

## Survey Statistics

In order to statistically analyze this survey, we need some information about you.

**21. I am ...**

- Male
- Female
- Divers

**22. I am.....years old**

**23. I live ...**

- With my biological parents
- With my mother and partner
- With my father and partner
- With my mother
- With my father
- With my grandparents or other relatives
- With my foster/adoptive parents
- In my own home
- In an institute
- In a group home
- Other, specifically:

**24. I have ...**

- No siblings
- 1-2 siblings
- 3 or more siblings

**25. In which region do you live?**

- Baden-Wuerttemberg

- Bavaria
- Berlin
- Brandenburg
- Bremen
- Hamburg
- Hesse
- Mecklenburg-Western Pomerania
- Lower Saxony
- North Rhine-Westphalia
- Rhineland-Palatinate
- Saarland
- Saxony
- Saxony-Anhalt
- Schleswig-Holstein
- Thuringia

**26. I live in a ...**

- Large city (with more than 500,000 inhabitants)
- Large city (with 100,000 to 500,000 inhabitants)
- Medium city (with 20,000 to 100,000 inhabitants)
- Small town (with 5,000 to 20,000 inhabitants)
- Rural community (with less than 5,000 inhabitants)

**27. I currently attend ...**

- An elementary school
- A junior high school
- A Secondary School/high school/comprehensive School
- A College
- No longer school

**28. After school I would like to ... / I am currently doing ...**

- Start an apprenticeship or vocational training
- Study at a university or a technical college
- Do voluntary service
- Do military service
- Doing nothing
- Other, specifically:

**Study director:** Prof. Dr. med. Jan de Laffolie
University Children's Hospital, Department of General Pediatrics and Neonatology, University Giessen, Giessen, Hesse, Germany

# CEDNA - The survey

as part of a study to improve the care of children and adolescents
with inflammatory bowel disease (IBD).

VERSION PARENTS - for parents of children and adolescents with IBD from 0 to 17 years

For **ease of reading,** the masculine, feminine, and various forms of the language are used interchangeably. All personal terms apply equally to all genders.

This survey is only for parents of children with inflammatory bowel disease (IBD) between the ages of 0 to 17. If you are a parent with multiple children with IBD, we ask that you complete a questionnaire for each child.

You can also **fill out CEDNA online**. See the info on the last page.

First, we will ask you to enter your own **questionnaire code,** because the survey is **anonymous**, which means that no one can can find out that you have filled it out. With the help of the code it is possible to filter out duplicate questionnaires in the scientific analysis.

Please enter your **code** here following the instructions:

| | | | | |
|---|---|---|---|---|
| ☐ | ☐ | ☐ | ☐ | ☐ |
| First letter of the first name of the patient's mother | Last letter of the first name of the patient | First letter of the patient's place of residence | First two digits of the birthday of the patient (e.g. 4 =04; 15=15) | |

## General questions about yourself

**01. How are you related to the IBD patient?**

- Mother
- Father
- Other, name:

**02. Have you been diagnosed with IBD?**

- Yes
- No

## Information about your child's illness

**03. Which IBD has your child currently been diagnosed with by a doctor?**

- Crohn's disease
- Ulcerative colitis
- Unclassified IBD

**04. Which concomitant diseases have been diagnosed in your child?** (multiple answers possible)

- Primary Sclerosing Cholangitis (PSC)
- Skin diseases (erythema nodosum, pyoderma gangraenosum)
- Eye diseases (e.g. uveitis, iridocyclitis)
- Gall and kidney stones
- Thrombosis
- Inflammatory joint diseases (e.g. rheumatism, idiopathic arthritis)
- Mental disorders (e.g. depression, anxiety disorder)
- Other, namely:
- None
- I do not know

**05. Please enter your child’s age.**

My child is......... years old.

**06. Please indicate your child’s gender.**

- Male
- Female
- Diverse

**07. How long has your child been diagnosed with IBD?**

- Less than 1 year
- 1 - 2 years
- 3 - 4 years
- 5 - 6 years
- More than 6 years

**08. In the past 12 months, who was your child's primary health care provider regarding IBD?** (Main contact person)

- No primary contact
- Primary care physician
- Pediatrician
- Pediatric gastroenterologist
- Adult Gastroenterologist
- Other, specifically:

**09. Looking at the course of your child's disease which of the following patterns of progression do you think is most likely to apply to your child?**

- After a few episodes of disease, there was a period of remission. This has continued to the present day
- There are repeated episodes of the disease, which are interrupted by resting phases
- Disease is constantly active, the symptoms vary in severity
- Disease becomes more active, the symptoms increase in severity

**10. The course of IBD is usually intermittent. Periods of no or minor symptoms (resting phase/remission) alternate with periodes of severe symptoms (acute symptoms / acute relapses). What phase of the disease is your child currently in?**

- Diagnosis phase
- Resting phase/remission
- Relapse phase
- I do not know

# Medical and psychological care

**11. If your child has a physical problem related to his or her IBD, can your child contact get the help he or she needs?**

- Yes, always ➞ Go to question 12
- Now and then ➞ Go to question 12
- Rather not ➞ Go to question 13

**12. Who can your child talk to about a physical problem related to his or her IBD?** (Multiple answers possible)

- Doctor
- Parents and family
- Friends
- Other, specifically:

**13. If your child has a mental/emotional problem related to his or her IBD: Can your child talk to someone who can give him/her the support he/she needs?**

- Yes, always ➞ Go to question 14.
- Sometimes ➞ Go to question 14
- Rather not ➞ Go to question 15

**14. Who can your child turn to for help with a mental or emotional problem in connection with his or her IBD?** (Multiple answers possible)

- Doctor
- Parents and family
- Friends
- Other, specifically:

**15. Do you have a pediatric gastroenterologist caring for your child?**

- Yes ➞ Go to question 16
- No ➞ Go to question 18

**16. How far do you travel to see a specialist and/or go to a specialty clinic where your child receives medical care?**

- Less than 30 minutes
- 30-60 minutes
- More than 60 minutes

**17. How many pediatric gastroenterologists and clinics specializing in children with IBD do you know of in your area (approx. 100 km away)?**

- None
- 1
- 2-5
- More than 5

**18. Are you generally satisfied with the medical care your child receives for IBD?**

- Not at all
- Only a little
- Mostly
- Very satiesfied

**19. Has your child ever been denied health insurance coverage because of IBD?**

- Yes, specifically:
- No

**20. Are you generally satisfied with the your child's health insurance coverage for IBD?**

- Yes
- No

# Managing inflammatory bowel disease

**We are interested in your child's treatment and management of inflammatory bowel disease. Please answer the following questions in relation to your child's current phase of the disease (phase of diagnosis, phase of acute relapse, phase of remission) of your child.**

**21. In the past three months, how often has your child used any of the following ways to cope with his or her own disease?**

| | never | rarely | regularly | often |
|---|---|---|---|---|
| Talking about IBD with others | | | | |
| Talking about IBD to family and/or friends | | | | |
| Talking about IBD with your doctor | | | | |
| Getting together with friends | | | | |
| Doing hobbies | | | | |
| Doing relaxation exercises (yoga, autogenic training, etc.) | | | | |
| Taking care of yourself physically | | | | |
| Exercise and be physically active | | | | |
| Pay special attention to the diet | | | | |
| Get psychological support | | | | |
| Participate in support group activities | | | | |
| Other, specifically: | | | | |

**22. During the current phase of your child's disease, how often have you felt the following emotions?**

| | Yes | No |
|---|---|---|

| | | |
|---|---|---|
| Anxiety | | |
| Shame | | |
| Uncertainty | | |
| Nervousness | | |
| Depressed mood | | |
| Serenity | | |
| Confidence | | |
| Helplessness | | |
| Excessive demands | | |
| Fatigue | | |
| Discouragement | | |
| Loneliness | | |
| Being left alone | | |
| Feeling guilty | | |
| Being overprotective | | |
| Fear for your child’s future | | |

**23. In the last three months, have you generally been able to cope with your feelings about your child's IBD?**

- ○ Not at all
- ○ Only a little
- ○ Most of the time
- ○ Totally

## How and where to find information

**The following questions are designed to help us understand how and where you find information about your child's disease. What are your ideas and wishes for finding and sharing information about IBD?**

**24. Do you feel adequately educated and informed about IBD?**

- ○ Not at all
- ○ Only a little
- ○ Mostly
- ○ Totally

**25. There are many topics related to IBD. Please indicate below how well informed you feel about each topic and if you would like to learn more.**

| **I feel well informed on ...** | **Not at all** | **Only a little** | **Mostly** | **Completely** | **I would like to know more** |
|---|---|---|---|---|---|
| IBD in general | | | | | |
| Causes of IBD | | | | | |
| Drug treatment options | | | | | |
| Surgical treatment options | | | | | |

| |
|---|
| Side effects of medications |
| Complications in the progressive<br>course of the disease |
| Concomitant diseases |
| Prognosis |
| Measures for preventive health care<br>(e.g. cancer screening, smoking cessation) |
| Complementary medicine<br>(e.g. homeopathy) |
| Vaccinations |
| Nutrition |
| Dealing with mental stress<br>and stress management |
| Psychotherapy |
| Transition to adult medicine (transition) |
| Sexuality issues |
| Family Planning |
| Social and legal issues<br>(Employment assistance, health insurance,<br>rehabilitation, law for the severely disabled,<br>pension insurance) |
| School and education<br>(e.g. compensation for disadvantages,<br>possibility to go to the toilet<br>at any time, information at school) |
| Patient organizations |
| Support groups |
| Traveling abroad with IBD |
| Other, specifically: |

**26. Please indicate which of the following treatment or counseling services you use or have used regarding your child's IBD. (Multiple answers possible)**

- Nutritional counseling
- Genetic counseling
- Counseling about having children
- Family counseling
- Marriage counseling
- Sexual counseling
- Physiotherapy
- Ergotherapy
- Psychological help/support
- Stress management services (e.g. relaxation training, autogenic training)
- Outpatient nursing services, home help
- Support groups
- Advice from health or long-term care insurance
- Advice from pension insurance (e.g. rehab)
- Event that provide information about the disease (e.g. attending a seminar)
- Transition programs (e.g. Berlin Transition Program)

- Other, specifically:
- I do not use or have not used any treatment or counseling services.

**27. Please indicate which of the following treatment or counselling services your child is using or has used  in relation to his or her IBD.** (Multiple answers possible)

- Nutrition counseling
- Genetic counseling
- Family counseling
- Sexual counseling
- Physical therapy
- Occupational therapy
- Psychological help/support
- Stress management services (e.g. relaxation training, autogenic training)
- Outpatient nursing services, home help
- Support groups
- Advice from health or long-term care insurance
- Advice from pension insurance (e.g. rehab)
- Events that provide information about the disease (e.g. attendance of a seminar)
- Transition programs (e.g. Berlin Transition Program)Other, specifically:
- My child does not use or did not use any treatment or counselling services.

**28. Please indicate how trustworthy you consider the following sources of information regarding IBD and whether you are aware of the source?**

| | **not at all** | **only a little** | **mostly** | **very much** | **I inform me about this source**<br><br>**Yes \| No** |
|---|---|---|---|---|---|
| Doctors | | | | | |
| Pharmacists | | | | | |
| Medical societies | | | | | |
| Pharmaceutical industry | | | | | |
| Health insurance companies | | | | | |
| Non-medical practitioners | | | | | |
| Nutritionists | | | | | |
| Psychologists | | | | | |
| Psychotherapists | | | | | |
| Transition programs | | | | | |
| Other patients | | | | | |
| Patient associations (e.g. DCCV) | | | | | |
| Support groups | | | | | |
| Family, friends, peers | | | | | |
| Specific Internet forums, chat rooms | | | | | |
| Internet in general | | | | | |
| Television, consumer programs | | | | | |
| Scientific journals, textbooks | | | | | |
| Politics | | | | | |

**29. How would you like to receive information about IBD?** (Multiple answers are possible)

**Printed information media**

- o Brochures and flyers
- o Age-appropriate books for children and teens
- o Professional journals and reference books
- o Other, specifically:

**Information events**

- o Lectures
- o Conferences
- o Other, specifically:

**Online information**

- o Internet sites
- o Patient counseling services
- o Educational films
- o Regular newsletters
- o YouTube channel
- o Blogs
- o Apps
- o Other, specifically:

**Online communication platforms**

- o Forums for patients and/or parents
- o Chats
- o Apps
- o Other, specifically:

**Informational seminars**

- o One-day workshops
- o Weekend seminar with overnight stay
- o Weekend seminar without lodging
- o Other, specifically:

**30. Who should provide you with information about IBD?** (Multiple answers possible)

- o Adult specialists (gastroenterologists)
- o Pediatric gastroenterologists
- o Nurses
- o Psychologists
- o Current research scientists

- Nutritionists
- Sports specialists
- Social workers
- Experiential and music educators
- Patient associations
- Support group representatives
- Affected families
- Affected adolescents
- Lawyers
- Representatives of consumer protection/help centers
- Health insurance companies
- Others, specifically:

**31. In the course of the disease there are different wishes and needs. The following list contains different topics. Please estimate which topic you would have been most interested in at the respective times of the IBD.**

| I would like information on ... | **at the time of diagnosis** | **in the first year** | **in the further course** | **I do not need any information** |
|---|---|---|---|---|
| IBD in general | | | | |
| Causes | | | | |
| Drug treatment options | | | | |
| Surgical treatment options | | | | |
| Side effects of medications | | | | |
| Complications in the course of the disease | | | | |
| Concomitant diseases | | | | |
| Prognosis | | | | |
| Preventive health measures (e.g. cancer screening, smoking cessation) | | | | |
| Complementary medicine (e.g. homeopathy) | | | | |
| Vaccinations | | | | |
| Nutrition | | | | |
| Coping with psychological stress and stress management | | | | |
| Psychotherapy | | | | |
| Transition to adult medicine (transition) | | | | |
| Sexuality issues | | | | |
| Family planning | | | | |
| Social and legal issues (e.g. employment promotion, health insurance, rehabilitation, pension insurance) | | | | |
| School and education (e.g. compensation for disadvantages, possibility to visit the toilet at any time, information at school) | | | | |

| |
|---|
| Patient organizations and self-help groups |
| Disease documentation (e.g. via IBD app) |
| Travel abroad |
| Other, specifically: |

**32. Now we are talking about information sharing opportunities. Would you attend one of the following? How many people should participate?**

| | Yes | No | 2- 5 | 6-10 | 11-15 | >15 |
|---|---|---|---|---|---|---|
| Group meeting of parents of affected children | | | | | | |
| Group meeting of affected children and teenagers | | | | | | |
| Group meetings of parents with their affected children | | | | | | |
| Family weekends for affected children, parents and siblings | | | | | | |
| Online communication platforms (e.g. forums, chats) | | | | | | |

## Survey Statistics

**In order to statistically analyze this survey, we would like to ask you to provide some information about yourself:**

**33. Please indicate your age.**

- ○ Younger than 20 years
- ○ 20-30 years
- ○ 31-40 years
- ○ 41-60 years
- ○ Older than 60 years

**34. In which family environment does your child live?**

- ○ In a family with two parents
- ○ With a single mother or father
- ○ Other, specifically:

**35. How many siblings does your child have?**

- ○ No siblings
- ○ 1-2 siblings
- ○ 3 or more siblings

**36. In which region do you live?**

- Baden-Wuerttemberg
- Bavaria
- Berlin
- Brandenburg
- Bremen
- Hamburg
- Hesse
- Mecklenburg-Western Pomerania
- Lower Saxony
- North Rhine-Westphalia
- Rhineland-Palatinate
- Saarland
- Saxony
- Saxony-Anhalt
- Schleswig-Holstein
- Thuringia

**37. What is the size of the city/town your child lives in?**

- Large city (with more than 500,000 inhabitants)
- Large city (with 100,000 to 500,000 inhabitants)
- Medium-sized city (with 20,000 to 100,000 inhabitants)
- Small town (with 5,000 to 20,000 inhabitants)
- Rural community (with less than 5,000 inhabitants)

**38. What is your highest school-leaving qualification?**

- No general school-leaving certificate or no school leaving certificate yet
- Secondary school leaving certificate
- Graduation from polytechnic secondary school
- Secondary school certificate or equivalent
- Grammar school

**39. What is your highest level of education?**

- No vocational qualification or still in vocational training
- Apprenticeship or dual vocational training
- Polytechnic degree (bachelor and/or master degree)
- University degree (bachelor and/or master and/or doctorate)

**40. Is your child's medical information already included in a IBD patient registry?**

- Yes
- No
- I do not know

**41. Which of the following medical and patient associations are you familiar with?**

- German Crohn's Disease / Ulcerative Colitis Association (DCCV e.V.)
- Society for Pediatric Gastroenterology and Nutrition (GPGE e.V.)
- Network for Children (KNW e.V.)
- German Society for Nutrition (DGE e.V.)
- German Self-Help Association for people with stoma and their relatives (ILCO e.V.)
- Competence Network for Bowel Diseases
- Transition Program (e.g. Berliner Transition Program)
- Others, specifically:

**Study director:** Prof. Dr. med. Jan de Laffolie
University Children's Hospital, Department of General Pediatrics and Neonatology, University Giessen, Giessen, Hesse, Germany
